# Oxidative DNA damage and total antioxidant status in glaucoma patients

**Published:** 2011-01-07

**Authors:** Rana Sorkhabi, Amir Ghorbanihaghjo, Alireza Javadzadeh, Nadereh Rashtchizadeh, Melorina Moharrery

**Affiliations:** 1 Drug Applied Research Center, Tabriz University of Medical Sciences, Tabriz, Iran; 2 Biotechnology Research Center, Tabriz University of Medical Sciences, Tabriz, Iran

## Abstract

**Purpose:**

To evaluate DNA damage markers and the antioxidant status of serum and aqueous humor in glaucoma patients.

**Methods:**

Aqueous humor and serum samples were obtained at the time of surgery from 28 patients with glaucoma and 27 patients with cataracts. Total antioxidant status (TAS) and 8-hydroxy-2´-deoxyguanosine (8-OHdG) levels of all samples were determined by spectrophotometric and enzyme-linked immunosorbent assay methods.

**Results:**

Aqueous levels of 8-OHdG were higher in glaucoma patients than in the cataract group (4.61±2.97 ng/ml versus 1.98±0.70 ng/ml, p=0.002). Serum levels of 8-OHdG were also higher in glaucoma patients than in the cataract group (17.80±8.06 ng/ml versus 13.63±3.54 ng/ml, p=0.046). The TAS levels of serum (0.55±0.13 mmol/lit versus 0.70±0.14, p=0.001), and aqueous humor (0.23±0.13 mmol/lit versus 0.34±0.15, p=0.001) in glaucoma patients were lower than in cataract patients.

**Conclusions:**

Our findings provide evidence that oxidative DNA damage increases and TAS decreases in the serum and aqueous humor of glaucoma patients. These findings support the hypothesis that the formation of reactive oxygen species and/or a decrease in TAS may have an important role in the pathogenesis of glaucoma.

## Introduction

Glaucoma is an insidiously progressive optic neuropathy which affects nearly 90 million people worldwide, and is the leading cause of irreversible blindness [1-3]. Funduscopic examination of the glaucomatous retina reveals characteristic excavation of the optic nerve head (ONH) with concomitant visual field defects [4]. It has been shown that the retinal ganglion cells (RGCs), which may die through an apoptotic process, lead to glaucomatous optic neuropathy [5-7]. In addition to elevated intraocular pressure (IOP) [8,9], retinal ischemia [10,11], nutritional status [12], and oxidative stress have been proposed as etiologic factors in the pathophysiology of glaucomatous RGC death [13-16]. Oxidative stress induced through the formation of multiple reactive oxygen species including superoxide (O_2_¯), hydrogen peroxide (H_2_O_2_), and hydroxyl radicals (OH) can initiate and propagate free radicals (FR) [17,18]. When FR levels increase and antioxidant defenses are insufficient, health problems may occur [19-21]. The net oxidative burden between these opposing pro-oxidant and antioxidant systems is the oxidative stress that damages lipids, proteins and DNA, culminating in cell death [22].

Oxidation of DNA is known to generate adducts of base and sugar groups, single strand and double-strand breaks in the backbone, and cross-links to other molecules. Among more than 20 known products resulting from DNA oxidation, 8-hydroxyl-2´-deoxyguanosine (8-OHdG) is easily quantified and commonly used to assess oxidative damage to DNA [23-27].

Recently, it has been demonstrated in vivo in humans that oxidative damage to DNA is significantly more abundant in the trabecular meshwork cells of glaucoma patients. Moreover, it has been shown in vivo in humans that both increased IOP and visual field damage are significantly related to the amount of oxidative DNA damage affecting TM cells [28,29].

Aqueous humor is known to contain several active oxidative agents, such as hydrogen peroxide and superoxide anion [30]. Both glutathione and ascorbate have been detected in aqueous humor. These antioxidants seem to play a particularly important role in glaucomatous disease [18]. The antioxidant status of biologic samples is regarded as an indicator of oxidative stress, and the measurement of TAS is one of the most commonly used and useful procedures to test for prediction of oxidative status [13]. To asses disturbance of the pro-oxidant/antioxidant balance, we evaluated the levels of 8-OHdG as an index of DNA oxidative damage and TAS as an predication index of oxidative status, as well as their correlations in the serum and aqueous humor of glaucoma patients.

The aim of the present study was to measure the DNA damage marker (8-OHdG) and the antioxidant status of the aqueous humor and serum in patients with glaucoma, and compare these with a control group. We also measured IOP and visual field indices such as mean deviation, pattern standard deviation, and cap to disc ratio in glaucoma patients, to explore whether there is any statistical correlation between the severity of disease and visual field defects and oxidative stress.

## Methods

### Study population

This prospective cross-sectional study was performed from September 2008 to May 2009 in the Glaucoma Service of Nikokari Eye Hospital in Tabriz, Iran.

Ethical approval was obtained from the Medical Ethics Committee of Tabriz University of Medical Sciences and written informed consent was received from all patients according to the tenets of the Declaration of Helsinki.

A total of 28 glaucoma patients and 27 cataract patients who were programmed for either glaucoma or cataract surgery were recruited. The inclusion criteria were based on 40–80 years old patients having primary open angle or pseudoexfoliative glaucoma or senile cataract. Both glaucoma and cataract patients with any of the following criteria were excluded: history of surgery, trauma, infection or inflammation, and systemic pathologies such as diabetes, renal and hepatic dysfunction, and uncontrolled hypertension (systolic blood pressure ≥140 mmHg, diastolic blood pressure ≥90 mmHg) . None of the subjects were smokers, had special diets, or were taking micronutrient supplements or antioxidant vitamins, such as α-tocopherol or ascorbic acid.

Glaucoma patients had either primary open angle glaucoma (n=15) or pseudoexfoliative glaucoma (n=13), and were scheduled for glaucoma filtering surgery or a combined procedure (phacotrabeculectomy). Among antiglaucoma medications, timolol, latanoprost, and dorzolamide were the most common drugs the glaucoma patients used before surgery.

All the patients enrolled in the cataract group had senile non-pathologic cataracts, did not have glaucoma, and were scheduled for phacoemulsification. The mean age was 65.54±9.43 for the glaucoma group and 65.22±9.79 years for the cataract group. All patients underwent a complete ophthalmologic evaluation that included medical history, slit lamp biomicroscopy, Goldmann applanation tonometry, and funduscopy. Additionally, patients with glaucoma underwent specific evaluation for glaucoma, such as gonioscopy and standard automated perimetry (SAP). SAP was performed with a Humphrey field analyzer HFA II (Carl Zeiss Meditec Inc., Dublin, CA) using a 30–2 threshold program with a standard Swedish interactive threshold algorithm (SITA) strategy.

### Sampling and primary tests

Aqueous humor samples (about 80 µl from each patient) were carefully collected at the beginning of surgery through paracentesis using a 27-gauge needle on a tuberculin syringe under an operating microscope, taking special care to avoid blood contamination. Aqueous humor was immediately cooled at −70 °C and transported to the laboratory to run the assays. Blood samples were obtained after overnight fasting one day before surgery. Serum samples were frozen immediately and stored at −70 °C until needed for analysis. All participants had normal renal and liver function as assessed by plasma urea, creatinine, alanine aminotranferase, and aspartate aminotransferase. Serum levels of total cholesterol, triglyceride, high-density lipoprotein cholesterol, and low-density lipoprotein cholesterol were determined using commercial reagents with an automated chemical analyzer (Abbott laboratories, Chicago, IL).

### 8-OHdG

The concentration of 8-OHdG in serum and aqueous humor was determined using an enzyme-linked immunosorbent assay; (Serum 8-OHdG check; Japan Institute for the Control of Aging, Shizuko, Japan). In brief, the anti-8-OHdG monoclonal antibody and the sample or standard were added to a microtiter plate precoated with 8-OHdG. An enzyme-labeled secondary antibody was used as a detection antibody.

### Total antioxidant status (TAS)

The TAS of samples was measured by spectrophotometric assay with a Randox total antioxidant status kit (LOT: 115813). In this method, incubation of 2,2´-azino-di(3-ethylbenzthiazoline sulfonate), ABTS, with a peroxidase (metmyoglobin) results in production of the radical cation ABTS^+^. This species is blue-green in color and can be detected at 600 nm. Antioxidants in the added sample inhibit this color production in proportion to their concentration.

### Statistical analysis

We used SPSS for Windows 13.0 (SPSS Inc., Chicago, IL). Data are presented as percentages and mean values ±SD. The independent *t*-test, Mann–Whitney U test, and logistic and regression tests were used as appropriate to assess the significance of differences between the two groups. Correlations between variables were evaluated using the Spearman test. In this study p≤0.05 was considered significant.

## Results

### Study Population

Fifty five patients, aged 40–80 years, were enrolled according to the inclusion/exclusion criteria mentioned. There were 28 patients with open angle glaucoma and 27 with non-pathologic senile cataracts. Table 1 summarizes the demographic characteristics of the study population. The two groups were matched for age and sex, and there were no differences in total cholesterol, triglyceride, high-density lipoprotein cholesterol, and low-density lipoprotein cholesterol levels between the groups.

**Table 1:** Demographic data for glaucoma and cataract patients.

**Variable**	**Glaucoma group (n=28)**	**Cataract group (n=27)**	p-value*
Age (years)**	66.54±9.43	65.22±9.79	0.86
**Sex**			
Male (%)	53.57	51.80	0.80
Female (%)	46.43	48.20	0.79
Lipid Profile**			
TG (mg/dl)	187.7±80.5	173.4±103.4	0.25
TC(mg/dl)	220.0±69.6	208.8±53.1	0.80
HDL-C(mg/dl)	48.8±8.4	49.0±10.3	0.29
LDL-C(mg/dl)	159.96±50.9	126.7±45.1	0.81
Oxidative stress markers**			
Serum TAS (mmol/l)	0.55±0.13	0.70±0.14	0.001
Aqueous TAS (mmol/l)	0.23±0.13	0.34 ±0.15	0.001
Serum 8-OHdG (ng/ml)	17.80±8.06	13.63±3.54	0.46
Aqueous 8-OHdG (ng/ml)	4.61±2.97	1.98±0.70	0.002

*Glaucoma versus Cataract group. **Data are reported as Mean±SD. In the table, TG: Triglyceride; TC: Total Cholestrol; HDL-CCT; High Density Lipoprotein Cholestrol; LDL-C; Low Density Lipoprotein Cholestrol; TAS: Total Antioxidant Status; 8-OHdG: 8-hydroxy- 2′- deoxyguanosine.

### Glaucoma patients

Table 2 shows some ophthalmic characteristics of the glaucoma patients. As indicated, there were no significant differences in clinical characteristics between the two subgroups; therefore all glaucoma patients were considered to be one group.

**Table 2:** Ophthalmologic characteristics of glaucoma patients expressed as mean ±standard deviation.

**Characteristics**	**POAG (n=15)**	**PXG (n=13)**	**p-value**	**Total**
IOP (mmHg) (preoperative)	28.47±6.34	24.87±6.14	0.48	26.67±6.40
Number of eye drops	2.73±0.70	2.80±0.41	0.90	2.77±0.57
Cup/Disc ratio	0.83±0.16	0.74±0.20	0.29	0.79±0.19
MD	−19.10±13.17	−21.61±6.87	0.06	−20.36±10.41
PSD	6.06±3.19	6.53±2.46	0.58	6.30±2.81

POAG: Primary Open Angle Glaucoma; PXG: Pseudoexfoliative Glaucoma; IOP: Intraocular pressure; MD: Mean Deviation; PSD: Pattern Standard Deviation.

### 8-OHdG

Aqueous levels of 8-OHdG were higher in glaucoma patients (4.61±2.97 ng/ml) than in the cataract group (1.98±0.70 ng/ml); p=0.002. Serum levels of 8-OHdG in glaucoma patients (17.80±8.06 ng/ml) were also higher than in the cataract group (13.63±3.54 ng/ml); p=0.046.

### TAS

The TAS values for the serum (0.55±0.13 mmol/lit versus 0.70±0.14, p=0.001), and aqueous humor (0.23±0.13 mmol/lit versus 0.34±0.15, p=0.001) were lower in glaucoma patients than in cataract patients.

### Correlations

The Spearman test showed good correlation among 8-OHdG levels and TAS in the serum (r=-0.76; p=0.00) and aqueous humor (r=-0.80, p=0.00) in glaucoma patients.

As shown in Table 3, glaucoma patients showed statistically significant correlation among serum and aqueous levels of TAS (r=0.48, p=0.01), but no correlation between serum and aqueous levels of 8-OHdG.

**Table 3:** Correlations between 8-OHdG and TAS in serum and aqueous humor of glaucoma patients.

** **	**8-OHdGs**		**8-OHdGa**		**TASs**		**TASa**	
**variable**	**r**	**p**	**r**	**p**	**r**	**p**	**r**	**p**
8-OHdGs	-	-	−0.21	0.29	−0.77	0.001	−0.17	0.40
8-OHdGa	−0.21	0.29	-	-	−0.20	0.31	−0.80	0.001
TASs	−0.77	0.001	−0.20	0.31	-	-	0.48	0.01
TASa	−0.17	0.40	−0.80	0.001	0.48	0.01	-	-

TASs: Total Antioxidant status of serum; TASa: Total Antioxidant status of aqueous; 8-OHdGs: 8-hydroxy- 2′- deoxyguanosine of serum; 8-OHdGa: 8-hydroxy- 2′- deoxyguanosine of aqueous.

Table 4 presents the correlation between 8-OHdG and TAS levels of serum and aqueous humor with clinical and perimetric parameters in the glaucoma group. We found no statistically significant correlations between these factors.

**Table 4:** Spearman correlation among ophthalmologic variables of glaucoma patients.

** **	**TASs**		**TASa**		**8OHdGs**		**8OHdGa**	
**Variable**	**r**	**p**	**r**	**p**	**r**	**p**	**r**	**p**
MD	−0.11	0.55	−0.09	0.61	0.11	0.95	0.20	0.29
PSD	−0.07	0.72	−0.16	0.40	0.06	0.76	−0.09	0.64
C/D ratio	0.7	0.69	−0.23	0.23	−0.00	0.98	0.04	0.81
Number of drops	−0.10	0.60	0.15	0.43	0.29	0.13	−0.35	0.06
IOP	−0.11	0.56	−0.01	0.96	0.02	0.91	0.01	0.93

MD: Mean Deviation; PSD: Pattern Standard Deviation, C/D ratio: Cup to Disc ratio; IOP: Intraocular pressure; TASs: Total Antioxidant status of serum; TASa: Total Antioxidant status of aqueous; 8-OHdGs: 8-hydroxy- 2′- deoxyguanosine of serum; 8-OHdGa: 8-hydroxy- 2′- deoxyguanosine of aqueous.

## Discussion

Although the pathogenic mechanism of glaucoma is not yet fully clarified, recent studies have demonstrated that oxidative damage constitutes an important pathologic step in inducing and maintaining the degeneration of the trabecular meshwork, optic nerve, and retinal ganglion cells [28].

Oxidative stress has been linked with cataracts for a long time [30-32]. We therefore chose cataract patients as a reference group to evaluate the oxidative stress in glaucoma patients.

The literature shows inconsistent findings regarding antioxidant activity in serum and aqueous humor in glaucoma patients. Yildrim and colleagues studied 40 patients with glaucoma and found no association between glaucoma and systemic myeloperoxidase or catalase enzyme activity [33]. In contrast, Gherghel and colleagues [34] concluded that glaucoma patients exhibit low levels of circulating glutathione, suggesting compromised oxidative defense. The only study of total reactive antioxidant potential (TRAP) and antioxidant enzymes in aqueous humor was performed by Ferreira and colleagues, and showed significantly decreased TRAP values and increased superoxide dismutase and glutathione peroxidase activity in glaucoma patients [13].

In the present study, we found significantly lower TAS levels in the serum and aqueous humor of glaucoma patients compared with cataract patients. We also found a significant correlation between aqueous and serum TAS levels in glaucoma patients. Although the findings in the literature and our study are contradictory, we think that the design and method of this study allow us to make a comment regarding this issue. Hence we speculate that decreased TAS levels may play a role in the occurrence and progression of glaucoma. It must be assumed that a decreased antioxidant capacity in tissues and body fluid may be the consequence of long lasting oxidative changes. For example, oxidative stress in the anterior chamber in our glaucoma patients may be an ocular manifestation of systemic disease. However, further randomized studies are needed to clarify this relationship.

The main targets of free-radical chain reactions are proteins, cell membranes, and DNA; particularly mitochondrial DNA. Indeed, mitochondrial DNA is less protected than nuclear DNA and therefore more sensitive to free radical attack [35,36]. A fairly significant correlation has been demonstrated by Sacca et al. between oxidative DNA damage in the human trabecular meshwork, increased intraocular pressure, and visual field defects in glaucomatous patients [37]. As far as we know, this is the first study to measure the levels of 8-OHdG as a DNA damage marker in the serum and aqueous humor of glaucoma patients. We found that the level of 8-OHdG in the aqueous humor of glaucoma patients was 2.1 fold higher than in the cataract group. In spite of the high aqueous to serum value of this marker, there was no statistically significant correlation between serum and aqueous levels for this marker. The findings support the hypothesis that in glaucoma the oxidative burden in the anterior chamber may be overwhelming, compromising the trabecular meshwork cells and their functions. This may be due more to a faulty antioxidative defense system and increased oxidative stress in the anterior chamber of glaucomatous eyes than to a systemic insult, and indicates that anterior segment structures are exposed to free radicals, suggesting that localized oxidative stress may contribute to the formation and development of glaucoma.

In contrast to the findings of Sacca et al. [37], no correlation was found in the present study between clinical or perimetric data and DNA damage markers in the serum and aqueous humor of glaucoma patients. This may be due to a decreased sensitivity and specificity of this marker in the serum and aqueous humor compared with trabecular meshwork tissue. This effect may have been compounded by the advanced stage of glaucoma in our patients, more than 70% of whom had a cup to disc ratio higher than 0.7.

A study by Zanon-Moreno et al. [38] showed a statistical difference in oxidative stress levels between patients with and without arterial hypertension. To eliminate this confounding effect, patients with uncontrolled hypertension were excluded from this study.

In the present study, the quantity and type of antiglaucoma eye drop instillation was taken into consideration. It has recently been suggested that topical antiglaucoma might increase oxidative stress, leading to programmed cell death. More than 90% of our glaucoma patients had received a combination of Timolol and Latanoprost before surgery. It has been suggested that prostaglandin agonists may induce apoptosis, but it seems that the effects are due more to the preservative benzalkonium chloride than to the antiglaucoma drug [39]. Other studies have also shown that timolol protects against free radical-induced apoptosis because of its antioxidant potential [40].We could find no correlation between the number of drugs and DNA damage markers in glaucoma patients, which may be due to the counterbalancing effect of the two main drugs most of our patients had taken.

We also need to mention some limitations of our study to guide future work. We firmly believe that glaucoma patients without topical antiglaucoma medication constitute an ideal study group, but could not include them due to ethical considerations.

Liu and coworkers [41], in an in-vitro study on cultured RGCs, demonstrated that oxidative stress is an early event in hydrostatic pressure or IOP induced neuronal damage. In an experimental rat study, Ozdemir and coworkers revealed that moderate IOP elevation could increase the generation of reactive oxygen species in the retina [42]. A further limitation of our study is the possibility that the oxidative stress and decreased antioxidant status observed may simply reflect ocular tissue changes secondary to elevated IOP, as nearly all of our patients were programmed for surgery due to high IOP.

In conclusion, the results support the belief that glaucomatous damage is the pathologic consequence of oxidative stress. An improved understanding of the role of serum and aqueous humor antioxidant status in the pathogenesis of glaucoma may change traditional glaucoma treatment, which is not always effective in protecting the optic nerve. Because of our assessment of oxidative stress and DNA damage markers in the aqueous humor, we cautiously propose that local damage in the anterior chamber occurs in glaucoma patients. We also believe that antioxidant drugs may be an option for these patients. However, further studies and clinical trials are needed to advance our understanding of the mechanisms of neuronal degeneration in glaucoma and find more effective therapies.
